# Electrocatalytic synthesis of ammonia by surface proton hopping†
†Electronic supplementary information (ESI) available. See DOI: 10.1039/c7sc00840f



**DOI:** 10.1039/c7sc00840f

**Published:** 2017-06-05

**Authors:** R. Manabe, H. Nakatsubo, A. Gondo, K. Murakami, S. Ogo, H. Tsuneki, M. Ikeda, A. Ishikawa, H. Nakai, Y. Sekine

**Affiliations:** a Department of Applied Chemistry , Waseda University , 3-4-1, Okubo, Shinjuku , Tokyo 169-8555 , Japan . Email: ysekine@waseda.jp; b Nippon Shokubai Co. Ltd. , 5-8, Nishiotabi, Suita , Osaka 564-0034 , Japan; c Department of Chemistry and Biochemistry , Waseda Univ. , Japan; d ESICB , Kyoto University , Kyoto-daigaku-katsura , Kyoto , 615-8520 Japan

## Abstract

Highly efficient ammonia synthesis at a low temperature is desirable for future energy and material sources. We accomplished efficient electrocatalytic low-temperature ammonia synthesis with the highest yield ever reported. The maximum ammonia synthesis rate was 30 099 μmol g_cat_^–1^ h^–1^ over a 9.9 wt% Cs/5.0 wt% Ru/SrZrO_3_ catalyst, which is a very high rate. Proton hopping on the surface of the heterogeneous catalyst played an important role in the reaction, revealed by *in situ* IR measurements. Hopping protons activate N_2_ even at low temperatures, and they moderate the harsh reaction condition requirements. Application of an electric field to the catalyst resulted in a drastic decrease in the apparent activation energy from 121 kJ mol^–1^ to 37 kJ mol^–1^. N_2_ dissociative adsorption is markedly promoted by the application of the electric field, as evidenced by DFT calculations. The process described herein opens the door for small-scale, on-demand ammonia synthesis.

## Abstract 2

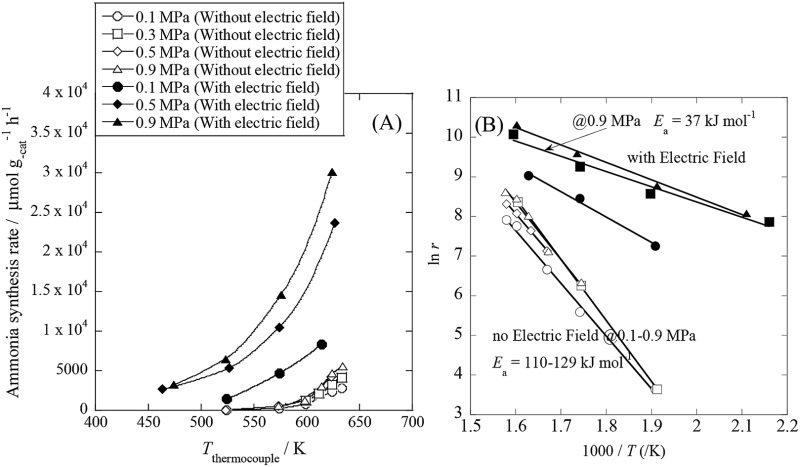
We accomplished efficient electrocatalytic low-temperature ammonia synthesis with the highest yield reported to date.

## Introduction

Ammonia is an important compound that is widely used as a raw material for chemical fertilizers, fibers, resins and refrigerants. Recently, ammonia has been suggested as a hydrogen carrier because of its high hydrogen content.^1^ At present, the Haber–Bosch process using nitrogen and hydrogen is the main method for ammonia synthesis. This process is conducted at high pressures (over 200 atm) and high reaction temperatures (around 773 K) because of its thermodynamic and kinetic limitations.^2^ For this reason, the energy consumption for ammonia synthesis is very large.^3^ Smaller scale, more dispersed ammonia plants could be developed if an ammonia synthesis route with milder operating conditions is realized. To date, numerous investigations into high-efficiency ammonia synthesis using milder operating conditions have been conducted. The original ammonia synthesis catalyst was an Fe-based double promotion catalyst.^4^ In 1972, Aika *et al.* reported a supported Ru catalyst^5^ that showed high activity for ammonia synthesis. This discovery of a Ru catalyst with alkali metals as co-catalysts^6–11^ led to a decrease in the reaction temperatures and pressures necessary for Haber–Bosch processing. Recently, Ru catalysts supported on praseodymium oxide,^12^ electride catalysts,^13–15^ and calcium amide^16^ have shown high ammonia synthesis rates, even at pressures and temperatures as low as 0.1 MPa and 600–700 K. In addition, ammonia synthesis at room temperature (around 298 K) and atmospheric pressure can be achieved using metal complexes including molybdenum,^17,18^ iron^19^ and cobalt,^20^ as well as with a titanium hydride compound.^21^ Moreover, examples of ammonia synthesis routes using an external DC electric field,^22^ plasma,^23–28^ photocatalysis^29^ and electrolysis^30^ have been reported, which indicate the possibility of low-temperature ammonia synthesis by exploiting the synergy between electrical/photochemical processes and catalysis. However, the ammonia synthesis rates using these methods are hindered by kinetic limitations. The present work proposes a new catalytic ammonia synthesis process assisted by an electric field which can show a high ammonia synthesis rate even at low temperatures. We achieved ammonia synthesis under very mild conditions (room temperature and pressures ranging from atmospheric up to 0.9 MPa) using a Ru–Cs catalyst. The process is more efficient than electrolytic synthesis because the process is non-faradaic (*λ* > 50). The process is also different from that using plasma, as it is conducted under much milder conditions with lower electric power consumption. This process is not intended as a replacement for the Haber–Bosch process, but rather for the creation of new uses and demand. Using this method of ammonia synthesis, highly pure ammonia can be collected as a compressed liquid by virtue of the extremely low reaction temperature. Therefore, small-scale and efficient processes for ammonia synthesis can be realized by application of an electric field.

## Results and discussion

### Kinetic analyses for catalytic ammonia synthesis in an electric field

**Fig. 1:**
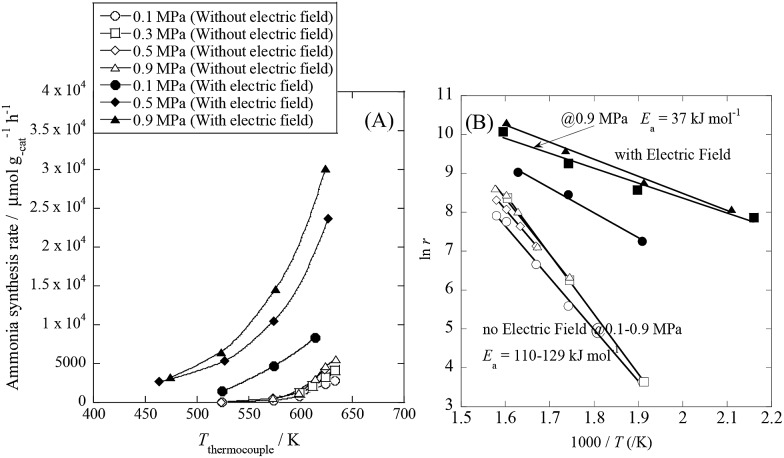
Temperature and pressure dependence of ammonia synthesis activity with or without an electric field. (A) Temperature dependence of the ammonia synthesis rate at various pressures, with or without an electric field. (B) Arrhenius plots for each reaction under various pressures in the kinetic regime: catalyst bed temperature, 463–634 K; catalyst, 9.9 wt% Cs/5.0 wt% Ru/SrZrO_3_, 200 mg; flow, N_2_ : H_2_ = 60 : 180 SCCM; current, 0 or 6 mA.

We conducted pre-screening tests for this purpose (results not shown), and we found that 9.9 wt% Cs/5.0 wt% Ru/SrZrO_3_ showed high activity for ammonia synthesis in an electric field, even at low reaction temperatures in the range of 463–634 K and pressures from atmospheric to 0.9 MPa, as presented in Fig. 1(A). We obtained a remarkably high ammonia yield, with an ammonia production rate as high as 30 099 μmol g_cat_^–1^ h^–1^ at 0.9 MPa, which is still in the kinetically controlled region. Interestingly, this impressive activity was also stable for 5 h. The application of an electric field increased the activity drastically, irrespective of the state of pressurization. The energy consumption was very low (only 2.82 W), and the ammonia production energy efficiency of 36.3 g kW h^–1^ is the highest ever reported. The faradaic efficiency (*i.e.* the ratio between the molar amounts of NH_3_ produced and electrons consumed) was as high as 26.88, strongly indicating that the reaction is non-faradaic. Interestingly, the pressure effects were more pronounced when the reaction proceeded under the influence of an electric field.

To elucidate the mechanism behind the electric field effect on catalytic ammonia synthesis, we conducted detailed kinetic analyses of the reaction. Fig. 1(B) presents Arrhenius plots for the ammonia synthesis reaction in the kinetic regime under both atmospheric and elevated pressure. The apparent activation energy decreased from 121 kJ mol^–1^ to 37 kJ mol^–1^ upon applying the electric field at 0.9 MPa. These results suggest that the ammonia synthesis reaction mechanism is affected by the electric field, and that the rate-determining step of the reaction changes.

To gain further mechanistic information, the N_2_, H_2_ and NH_3_ pressure dependencies of the ammonia synthesis rate were investigated. The obtained results are presented in Table S1 of the ESI.† With the assumption that the rate of the ammonia synthesis reaction can be written as in eqn (1), eqn (2)–(5) were used for kinetic analyses:^31,32^

1 *r* = *kP*_N_2__^*α*^*P*_H_2__^*β*^*P*_NH_3__^*γ*^

2 *r* = *W*^–1^d*y*_0_/d(1/*q*)

3 log *y*_0_ = log(*c*/*q*)^1/*m*^

4 *r* = *W*^–1^*c*/*my*_0_^(1–*m*)^

5 *c* = *k*′*P*_N_2__^*α*^*P*_H_2__^*β*^,

where *r* stands for the reaction rate of the ammonia synthesis, *W* denotes the catalyst weight, *y*_0_ signifies the ammonia mole fraction, *q* represents the mass flow and (1 – *m*) corresponds to *γ*.

As shown in Table S1,† for the catalytic reaction (without the electric field), the ammonia synthesis rate exhibited a positive N_2_ partial pressure dependence with an order of 0.68. However, the H_2_ and NH_3_ partial pressure dependencies of the reaction rate were negative (–0.21 for H_2_ and –0.1 for NH_3_). These trends are in excellent agreement with past kinetic studies of ammonia synthesis.^8,11,32,33^ They indicated that the rate-determining steps of the catalytic reaction without the electric field include N_2_ activation, especially N_2_ dissociative adsorption on Ru, because of the strong triple bond energy of N_2_. However, when the catalyst included an electron donor such as Cs, the reaction order of N_2_ became smaller than unity, at around 0.7.^8^ In addition, the negative H_2_ pressure dependence indicates that the surface of Ru was poisoned by hydrogen. On the other hand, upon application of the electric field, the N_2_ and H_2_ pressure dependencies of the ammonia synthesis rate changed drastically. The N_2_ pressure dependence of the reaction rate decreased upon applying the electric field, indicating that the N_2_ activation steps were promoted by the electric field. In addition, the H_2_ pressure dependence of the reaction rate weakened; the reaction orders were 0.24 for N_2_, 0 for H_2_ and –0.26 for NH_3_. Therefore, the poisoning of Ru by hydrogen disappeared to some extent upon application of the electric field.

**Fig. 2:**
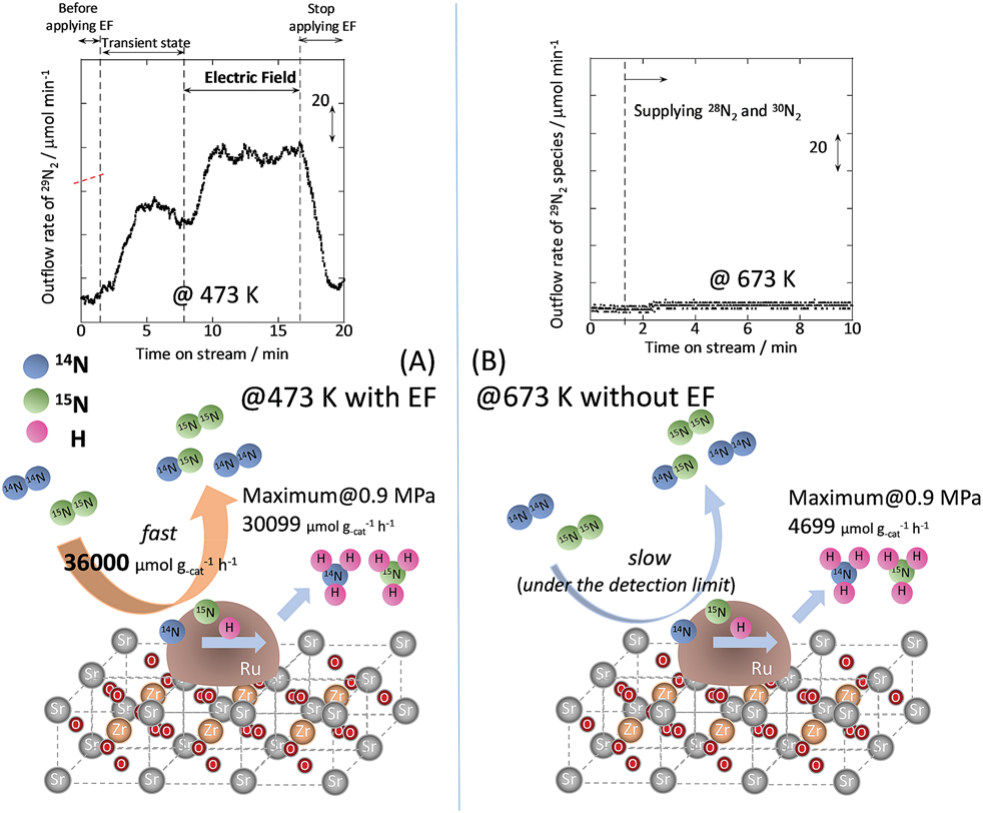
Isotope exchange tests using ^30^N_2_, (A) with an electric field at 473 K, and (B) without an electric field at 673 K. Catalyst, 9.9 wt% Cs/5.0 wt% Ru/SrZrO_3_, 200 mg; flow, ^28^N_2_ : ^30^N_2_ : H_2_ : Ar = 6 : 6 : 36 : 12 SCCM; current, 0 or 6 mA.

To specifically examine the effect of the electric field on N_2_ activation, isotope exchange tests were conducted using ^30^N_2_. Fig. 2 presents the results obtained in transient response tests using ^28^N_2_ and switching to ^30^N_2_. When ^30^N_2_ was supplied in the presence of the electric field at 473 K, ^29^N_2_ formation was observed. However, without the electric field, ^29^N_2_ was not detected, even at higher reaction temperatures, as shown in Fig. 2 (*T* ≤ 673 K). Generally, the isotope exchange tests using ^30^N_2_ were conducted while only supplying N_2_ species to avoid H_2_ poisoning of the Ru surface.^34^ In our isotopic tests without supplying H_2_ and without application of the electric field, ^29^N_2_ was not detected either, even at 873 K (Fig. S1†). In addition, H_2_ was necessary for the isotopic tests when applying the electric field because without a hydrogen supply, application of the electric field resulted in a spark discharge. This phenomenon demonstrates that hydrogen, or a chemical compound containing hydrogen, plays the role of an ion carrier, enabling the stable application of the electric field.

As shown in eqn (6), ^29^N_2_ was produced through the dissociative adsorption of ^28^N_2_ and ^30^N_2_, followed by the recombination of ^28^N_2_ and ^30^N_2_. The N_2_ dissociation rate can therefore be calculated from the ^29^N_2_ production rate, using eqn (7)–(9), assuming steady-state conditions at the surface of the catalyst:^34,35^

6 ^28^N_2_ + ^30^N_2_ → 2^29^N_2_

The balance equation for N species flow is

7 *V*_in_ – *V*_out_ – *r*_NH_3__/2 = 0

and the equations for N_2_ outflow are

8 *F*_28_ = (*F*_28_)_0_ + *V*_out_ × (*f*_s14_*f*_s14_) – *V*_in_ × (*f*_28_)_0_

and

9 *F*_29_ = *V*_out_ × (2*f*_s14_*f*_s15_) = *V*_out_ × {2*f*_s14_ × (1 – *f*_s14_)},

where *V* signifies the total flow rate, *F* denotes the flow of each species, and *f* represents the mole fraction of the isotopic species. Subscripts 28, 29, 14 and 15 respectively denote ^28^N_2_, ^29^N_2_, ^14^N and ^15^N, subscript s signifies the Ru surface, and subscript 0 denotes an input value (detailed procedures are presented in the ESI†). From the calculation, the N_2_ dissociation rate per gram of catalyst was about 36 000 μmol g^–1^ h^–1^ when applying the electric field at 473 K. However, the N_2_ dissociation rate could not be calculated for the catalytic reaction because ^29^N_2_ was below the limit of detection. These results indicate that N_2_ dissociative adsorption is very rapid and is enhanced irreversibly in the electric field.

### *In situ* DRIFTS measurements

**Fig. 3:**
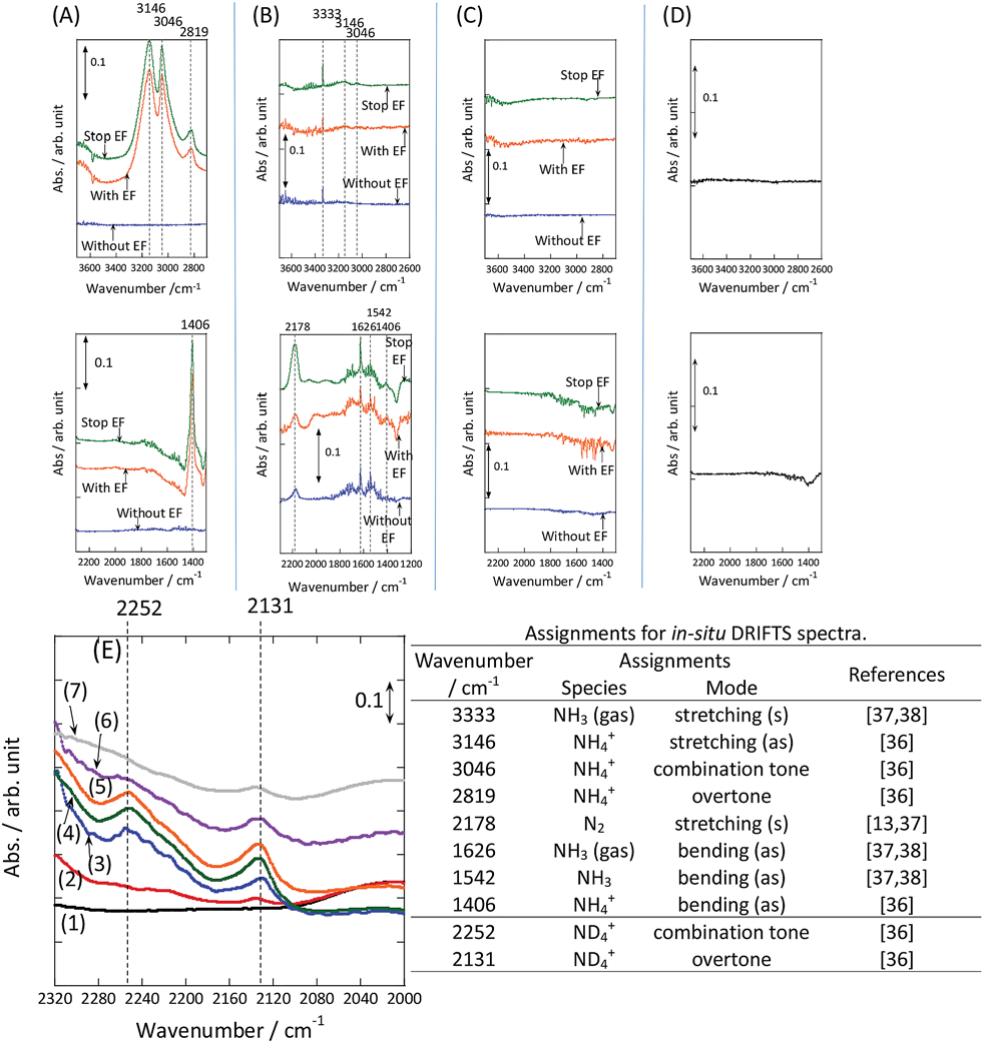
*In situ* DRIFTS spectra with, without, and after switching off the electric field (EF). (A) N_2_ : H_2_ = 15 : 45 SCCM at 473 K; (B) 10% NH_3_/He : Ar = 1 : 59 SCCM at 473 K; (C) N_2_ : Ar = 15 : 45 SCCM at 473 K; (D) N_2_ : H_2_ = 15 : 45 SCCM at 648 K (without the EF, catalytic reaction); (E) experiments using an isotope (D_2_) at 473 K: (1) after imposing the EF with N_2_ and H_2_, (2) when D_2_ (15 SCCM) was supplied, (3) when D_2_ was supplied with the EF (10 mA), (4) after imposing the EF with D_2_, (5) when H_2_ (15 SCCM) was supplied again, (6) when H_2_ was supplied with the EF (10 mA), (7) after imposing the EF with H_2_ and the catalyst (9.9 wt% Cs/5.0 wt% Ru/SrZrO_3_; current, 0, 6, or 10 mA).

To observe the adsorbed species on the surface of the catalyst during application of the electric field for ammonia synthesis, and to reveal the mechanism of the ammonia synthesis reaction in the presence of the electric field, *in situ* diffuse reflectance infrared Fourier transform spectroscopy (DRIFTS) measurements were conducted. With a customized cell, we measured the IR spectrum when the electric field was applied to the catalyst bed, as presented in Fig. S2.† The obtained *in situ* DRIFTS spectra under each condition are shown in Fig. 3. Assignments are presented in the table in Fig. 3. As shown in Fig. 3(A), no peaks were observed when supplying only N_2_ and H_2_ at 473 K. However, when applying the electric field, four sharp peaks around 3146, 3046, 2819 and 1406 cm^–1^ were detected. These peaks were not observed under a pure N_2_ supply with the electric field (C), or during ammonia synthesis at 648 K without the electric field (D). Therefore, these four peaks were observed only when the electric field was applied to the catalyst bed. They were assigned to the stretching, combination tone, overtone and bending modes of N–H vibrations derived from NH_4_^+^.^36^ In addition, these species remained on the catalyst surface after cessation of the electric field. Presumably, NH_4_^+^ was produced from synthesized NH_3_ and protons. The protons are generated from H_2_ and hop on the catalyst surface when the electric field is applied. The ammonium cation only exists in a stable form on the catalyst. Fig. 3(E) shows the *in situ* DRIFTS spectra with D_2_. The peaks derived from the isotopes did not appear merely by supplying D_2_ to the produced ammonium ions (spectrum 2). However, upon application of the electric field, two peaks assigned to the combination tone and overtone modes of ND_4_^+^ were observed at around 2252 and 2131 cm^–1^ (spectrum 3).^36^ These peaks were weakened under H_2_ flow conditions in the presence of the electric field (spectrum 6). Based on the above observations, the protons are considered to hop *via* NH_4_^+^ ions and the catalyst support when the electric field is applied to the catalyst bed. The electric field could not be applied without supplying H_2_. Fig. 3(B) shows the *in situ* DRIFTS spectra while supplying NH_3_ at 473 K. Peaks derived from NH_3_ and N_2_ species were also detected at around 3333, 1626, 1542 and 2178 cm^–1^.^13,37,38^ However, peaks assignable to the N–H vibrations derived from NH_4_^+^ were very weak compared with those in Fig. 3(A), even when applying the electric field. These results show that surface protonics occur only when the forward reaction for ammonia synthesis proceeds in the presence of an electric field.

### Theoretical calculations for ammonia synthesis with/without the electric field and the proposed mechanism of ammonia synthesis in the electric field

**Fig. 4:**
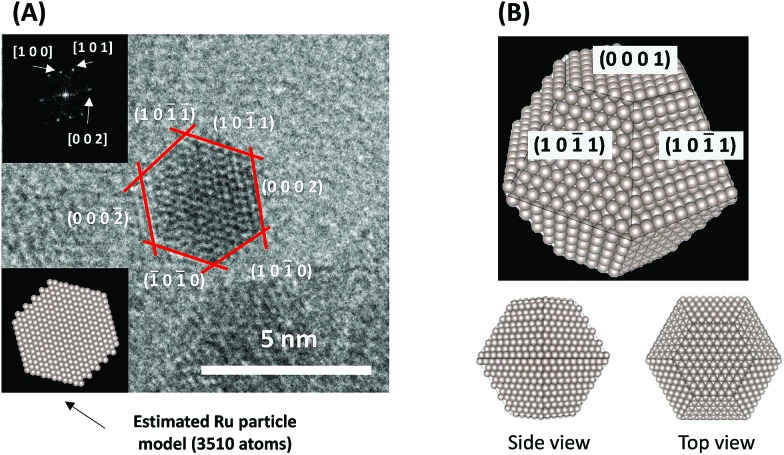
(A) Representative TEM images of a Ru particle supported on SrZrO_3_ and (B) proposed models for the Ru particles.

To consider the reaction mechanism experimentally and theoretically, we considered the (0001) and (1011) facets, which are the exposed facets at the surface of the Ru particles, as observed by the transmission electron microscope (TEM) images in Fig. 4.

To examine how the effect of an electric field on the Ru catalyst can be expressed by theoretical computation, the *in situ* IR spectra of CO adsorbed on Ru/SrZrO_3_ were measured (Fig. S3–S6†). The IR spectra with and without an electric field indicate that a blue shift of the CO vibrational frequency occurred when an electric field was applied to the Ru catalyst. Using density functional theory (DFT), the blue shift of the CO vibrational mode was observed to occur when positive charge was introduced into the system, irrespective of the facet and adsorption site (Table S2†). Thus, these results suggest that the effect of an electric field on Ru is well expressed by introducing positive charge into the system.

Next, the ammonia synthesis reaction under an applied electric field was theoretically considered. As suggested by the experimental results, the ammonia synthesis reaction under an applied electric field is significantly different from the conventional catalytic ammonia synthesis reaction, because in the former, protonics govern the reaction. Considering this, both the “dissociative mechanism”,

10 N_2_ + 2* → 2N*

11 H_2_ + 2* ⇆ 2H*

12 N* + H* ⇆ NH* + *

13
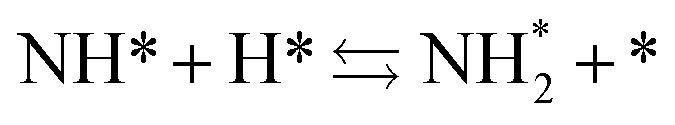

14
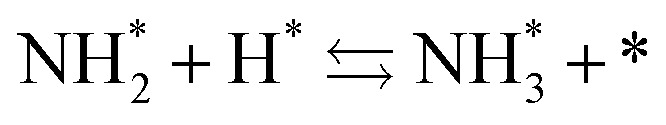

15
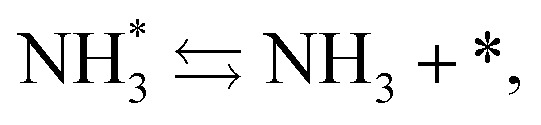
and the “associative mechanism”,

16 H_2_ + 2* ⇆ 2H*

17 N_2_ + H* → N_2_H*

18 N_2_H* ⇆ NH* + N*

19
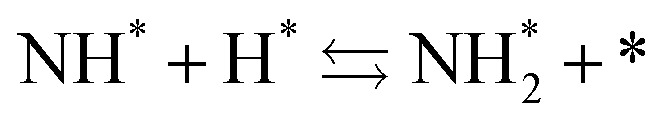

20
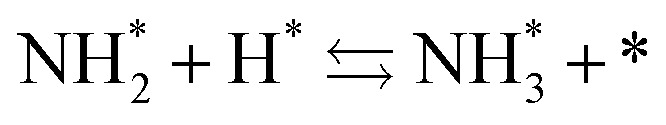

21
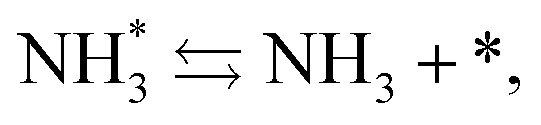
were examined using theoretical calculations.^39–42^ Here, the asterisk (*) denotes a vacant surface site, and a species with an asterisk is an adsorbed species. The rate-determining steps of the dissociative and associative mechanisms were found to be N_2_ dissociation and N_2_H formation, respectively; thus the activation barriers and reaction energies for these two steps were examined.

**Fig. 5:**
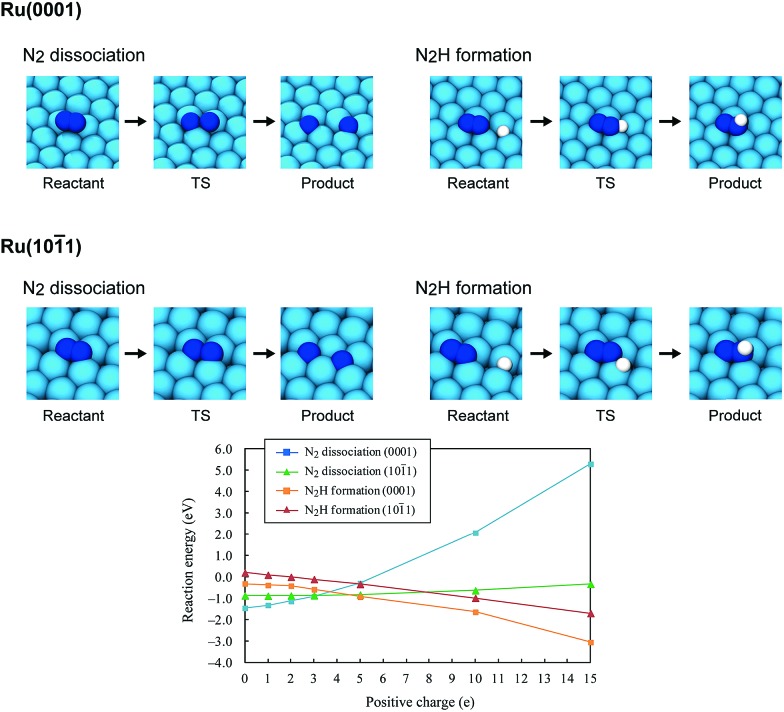
Optimized structures of the reactant state, transition state (TS) and product state of the N_2_ dissociation and N_2_H formation reactions on Ru(0001) and Ru(1011), the reaction energies of the N_2_ dissociation and N_2_H formation reactions on Ru(0001) and Ru(1011), and their dependence on the electric field strength, expressed by the magnitude of the positive charge. On Ru(0001), the dissociated N atoms occupy fcc and hcp hollow sites, and N_2_H formation generates N_2_H adsorbed on hcp sites. On Ru(1011), both the dissociated N atoms and generated N_2_H occupy four-fold hollow sites.

Using the DFT method, the reaction energies (Δ*E*) of the N_2_ dissociation and N_2_H formation reactions were calculated (Fig. 5). The results show that the application of an electric field, which is simulated by the positive charge in the system, induces an increase in Δ*E* for N_2_ dissociation but a decrease in Δ*E* for N_2_H formation. As a result, without an electric field, the formation of N_2_H is an endothermic process but it becomes an exothermic process under an applied electric field. The calculated activation energies (*E*_a_) of these processes (Fig. S7(A)†) also indicate that N_2_H is more easily formed when an electric field is applied; *E*_a_ significantly decreased from 0.93 eV (N_2_ dissociation) to 0.61 eV (N_2_H formation) when 15 positive charges were introduced into the computational model. Here, the *E*_a_ values on Ru(1011) were used because this surface has a larger area in the assumed Ru particle (Fig. 4). This explains the significant decrease of the activation energy with the application of an electric field.

In addition to the activation energy, the experimental Arrhenius plot in Fig. 1(B) suggests that the application of an electric field causes a large decrease in the pre-exponential factor. Interestingly, based on transition state theory, the pre-exponential factors for the dissociative and associative mechanisms are very different because they are calculated as22
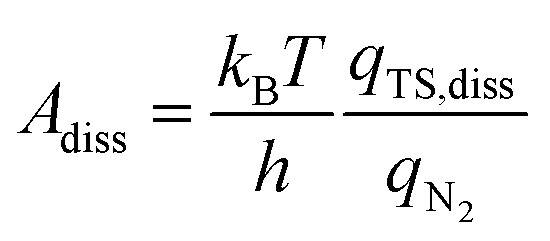
and23
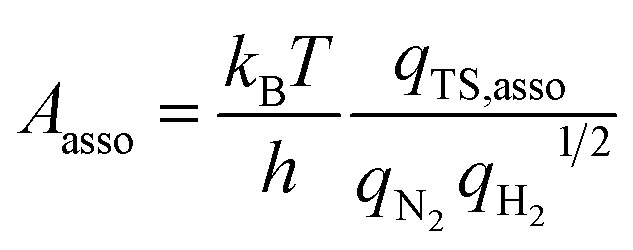
respectively, where *k*_B_ and *h* are the Boltzmann and Planck constants, *q*_N_2__ and *q*_H_2__ are the molecular partition functions for N_2_ and H_2_, and *q*_TS,diss_ and *q*_TS,asso_ are the molecular partition functions for the transition states of the dissociative and associative mechanisms. The presence of *q*_H_2__ in the denominator of eqn (23) makes *A*_asso_ smaller than *A*_diss_ by 4.5 × 10^2^.

Based on these pre-exponential factors and calculated activation energies, a theoretical Arrhenius plot can be constructed (Fig. S7(B)†). Our theoretical plot can explain two important differences between the experimental plots with and without an electric field, when an associative mechanism is assumed for the ammonia synthesis reaction under an applied electric field. Thus, our calculation assuming the associative mechanism explains both the large decreases in *E*_a_ and the pre-exponential factor. This strongly suggests that the ammonia synthesis reaction with and without an electric field proceeds *via* different mechanisms, *i.e.* dissociative and associative mechanisms, respectively.

**Fig. 6:**
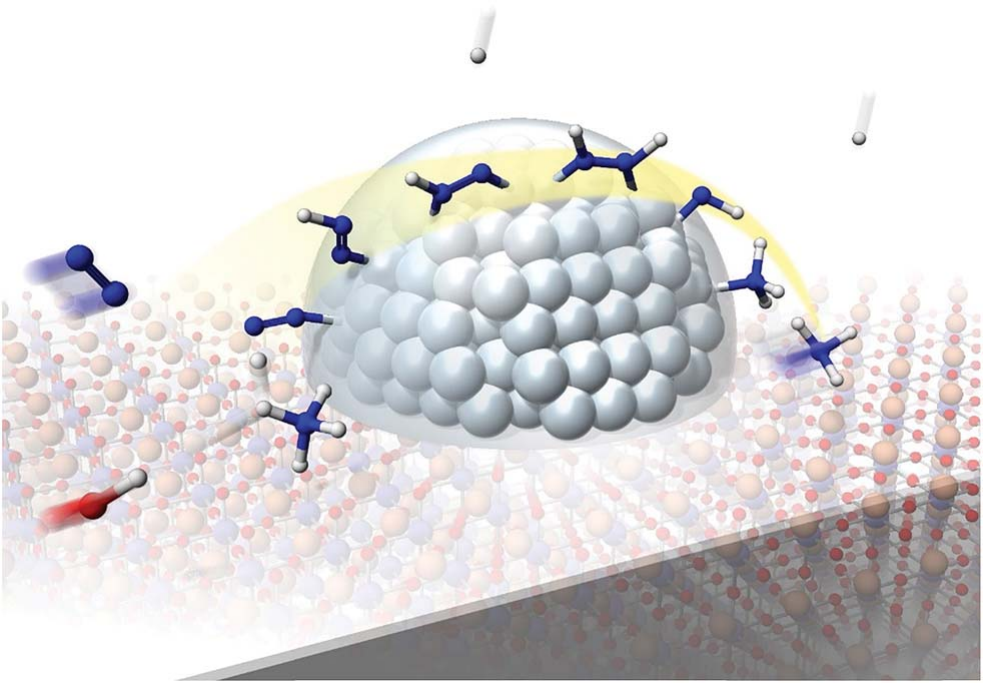
Schematic illustrating the mechanism of the ammonia synthesis reaction in an electric field.

Our experimental and theoretical investigations demonstrated that proton hopping in an electric field on the catalyst surface plays an important role in N_2_ activation at low temperatures. The proposed mechanism for ammonia synthesis in an electric field is presented in Fig. 6. The peculiar surface conduction caused by the electric field, proton hopping, is considered to enable ammonia synthesis to proceed at low temperatures and atmospheric pressure.

## Conclusions

Ammonia synthesis was performed over a 9.9 wt% Cs/5.0 wt% Ru/SrZrO_3_ catalyst in an electric field under various pressures. The ammonia synthesis activity of this catalyst increased drastically upon application of the electric field under both atmospheric and 0.9 MPa pressure, even at low reaction temperatures. The maximum ammonia yield per gram of catalyst was 30 099 μmol g^–1^ h^–1^, which is the highest value reported to date. To elucidate the effects of the electric field on ammonia synthesis, a kinetic investigation and *in situ* DRIFTS measurements were conducted under atmospheric pressure. The results of our kinetic analyses demonstrated that both the apparent activation energy and the dependence of the reaction rate on N_2_ pressure decreased upon application of the electric field, indicating that clearly different reaction mechanisms occur with and without application of the electric field. Furthermore, isotope exchange tests demonstrated that N_2_ dissociative adsorption was markedly promoted by application of the electric field. The *in situ* DRIFTS results revealed that proton conduction *via* NH_4_^+^ and the catalyst support occurred when the electric field was applied. These unique surface protonics are strongly associated with N_2_ activation, which moderates the severe conditions required for ammonia synthesis. The mechanism of ammonia synthesis in an electric field proposed here was also supported by theoretical calculations, demonstrating that the mechanism changed from dissociative to associative upon application of the electric field. In summary, surface protonics induced by the application of an electric field have an important role to play in the enhancement of catalytic ammonia synthesis under mild conditions.
