# Supplementary material for: Electrocatalytic synthesis of ammonia by surface proton hopping
Source: Chem Sci. 2017 Jun 5;8(8):5434–9. doi: 10.1039/c7sc00840f (PMC5609515; doi:10.1039/c7sc00840f)
Supplement: Supplementary file 1 [file SC-008-C7SC00840F-s001.pdf]

Electronic Supplementary Material (ESI) for Chemical Science.
This journal is © The Royal Society of Chemistry 2017

Electro-catalytic synthesis of ammonia by surface proton hopping

R. Manabe[a], H. Nakatsubo[a], A. Gondo[a], K. Murakami[a], S. Ogo[a], H. Tsuneki[b], M. Ikeda[b], A. Ishikawa[c], H. Nakai[c,d], Y. Sekine*[a]

ESI (Electronic Supplementary Information)

Methods

Preparation for catalyst supports. Various oxides (CeO_2, ZrO_2, $Ce_{0.5}Zr_{0.5}O_2$, $SrZrO_3$, and $SrCeO_3$) are used as catalyst supports. Catalyst supports were prepared using a complex polymerization method. First, ethylene glycol and citric acid were measured in an amount of 3 mol of metallic amount, and dissolved in distilled water. Then, metal nitrate ($Ce(NO_3)_2$ ($6H_2O$), $ZrO(NO_3)_2$ ($2H_2O$), and/or $Sr(NO_3)_2$) were dissolved in the solution and stirred. Then the obtained solution was heated and stirred at 343 K. The obtained sample was pre-calcined at 673 K for 2 h. Subsequently, it was calcined under air flow at 1123 K for 10 h.

Preparation for catalysts. Using prepared supports, Ru-loaded catalysts (Ru/Oxide) were prepared for this work using an impregnation method for this work. $RuCl_3$ ($3H_2O$) or $Ru(acac)_3$ was used as the metal precursor. First the distilled water solvent with prepared support oxide was evaporated *in vacuo* at room temperature for 2 h. Then the solvent with Ru precursor was added and stirred for another 2 h. The obtained solution was heated and stirred at 343 K, and dried at 393 K for 20 h. The dried sample was treated under H_2 (50 SCCM) and Ar (50 SCCM) flow at 723 K for 2 h. The Ru content was fixed at 5.0 wt%. Cesium-promoted Ru catalysts were prepared using the sequential impregnation method. The solvent of Ru-loaded catalyst was evaporated at room temperature *in vacuo* for 2 h. Then $CsNO_3$ aqueous solution was added to the impregnated sample and stirred for another 2 h. The solvent was heated and stirred at 343 K and then dried at 393 K for 20 h. The amount of Cs was 0, 3.3, 6.6, 9.9, and 13.2 wt%. The prepared catalyst was crushed into 355–500 μm diameter particles.

Catalytic activity tests. In all activity tests, a quartz tube (6.0 mm i. d.) was used for a fixed-bed flow-type reactor as shown in supplementary materials figure S8. Two stainless steel rods (6 or 2

mm o. d.) were inserted into the reactors as electrodes. The upper electrode was set on the top of the catalyst bed. The ground electrode was set on the bottom of the catalyst bed. The catalyst bed temperature can be measured using a thermocouple. The imposed current and response voltage waves were observed using a digital phosphor oscilloscope (TCP A 300, and TDS 2001C; Tektronix Inc.). Before the reaction, the catalyst was pre-reduced for activation under N_2 (60 SCCM) and H_2 (180 SCCM) flow at 723 K for 2 h. Activity tests were conducted basically with 200 mg catalyst under N_2 (60 SCCM) and H_2 (180 SCCM) flow at various furnace temperatures, 6 mA (D.C.). Product gases were analyzed by using a gas chromatograph-TCD. The trapped ammonia was analyzed using an ion chromatograph (IC-2001, Tosoh Corp.). When using isotopes, $^{30}N_2$ (6 SCCM) was introduced with $^{28}N_2$ (6 SCCM), H_2 (36 SCCM), and Ar (12 SCCM). To detect $^{28}N_2$, $^{29}N_2$, and $^{30}N_2$, Q-Mass (QGA; Hiden Analytical Ltd.) was used for qualitative and quantitative analyses.

***In-situ* Diffuse Reflectance Infrared Fourier Transform Spectroscopy (DRIFTS) measurements.** To elucidate the adsorbed species on catalysts with and without the electric field, *in-situ* DRIFTS measurements were conducted using FT-IR (FT-IR6200; Jasco Corp.) with an MCT detector and a diffuse reflectance infrared Fourier transform spectroscopy reactor cell (DR-600Ai; Jasco Corp.) with the ZnSe window. For IR measurements with application of the electric field, two DRIFTS cells were used. They were made of SUS304 for catalytic reaction (for tolerance of high temperatures) and of Teflon for catalytic reaction in the electric field (to avoid short circuits in the cell), as presented in supplementary materials figure S2. The sample was 9.9 wt%Cs / 5.0 wt%Ru / $SrZrO_3$. First, the sample was pre-reduced under N_2 (15 SCCM) and H_2 (45 SCCM) flow at 473 or 723 K for 2 h. Then, the reducing gas was purged for 30 min with Ar (60 SCCM). Then background (BKG) measurements were taken under Ar gas at 473 K or 648 K. Subsequently, the reactant gas (N_2 : H_2 = 15 : 45 SCCM or N_2 : Ar = 15 : 45 SCCM or 10%NH_3/He : Ar = 1 : 59 SCCM, or H_2/D_2 = 15 SCCM) was supplied for 30 min at most. Subsequently, the electric field was applied for about 10 min. Each spectrum was recorded at resolution of 4.0 cm^{-1}, over 50 scans. The imposed current was 6 or 10 mA.

Characterization of catalyst. The crystalline structure was characterized using powder X-ray diffraction (XRD, SmartLab3; Rigaku Corp.) operating at 40 kV and 40 mA with Cu-*K*α radiation. The XRD measurement results are presented in supplementary materials figure S9. The dispersion

ratio and particle diameter of Ru catalysts are characterized using CO pulse (BEL CAT II; Bel Japan, Inc.). Before measurements, the catalyst sample was pre-treated under H_2 (50 SCCM) flow at 723 K for 30 min. After the treatment, the temperature was decreased to 323 K with He, and 10% CO was pulsed. Results of the CO pulse are presented in supplementary materials table S3. The structure of supported Ru was observed using a TEM (JEM-2100F, 200kV).

Theoretical calculations for ammonia synthesis on Ru with/without the electric field. All the calculations were conducted using the Vienna *ab initio* simulation package (VASP) 5.4. A core-valence effect was included with the projector-augmented wave (PAW) method [43, 44]. The valence part wave functions were expanded using plane-wave basis sets with kinetic energy lower than 400 eV. For the calculation of energetics, i.e. adsorption energy, reaction energy, and activation barriers, re-parameterized Perdew-Burke-Ernzerhof (RPBE) was used as the exchange-correlation functional for spin-polarized DFT calculation [45, 46]. For vibrational frequency calculations, PW91 exchange-correlation functional was used [47]. The DFT calculations were done in a spin-polarized manner. For *k*-space, integration was conducted by taking 3 × 3 × 1 points. For the smearing method in the electronic state, the first-order Methfessel-Paxton method with $\sigma = 0.2$ and tetrahedron method were used, respectively, for catalyst system and free-molecules (N_2, H_2, etc.). Both Ru(0001) and Ru(101) surfaces were modeled by four Ru atomic layers. During geometry optimization, the lower and upper two layers of Ru were fixed and relaxed, respectively. The geometry of adsorbates was fully relaxed. The catalytic system was represented by repeated slabs, which were separated by an approximately 20 Å vacuum layer. The definitions of adsorption sites on Ru(0001) and Ru(101) are presented in supplementary materials figure S10. For a transition state search, nudged elastic band (NEB) method was used. The electric field effect was considered by adding or removing electrons from the model system. Monopole and dipole corrections were included. Vibrational frequencies of CO were obtained by diagonalizing the Hessian matrix, which was computed from a finite-difference method. The C-O stretching frequencies perpendicular to the surface were calculated.

Apparatus and IR cell for applying electric field to catalyst bed. We prepared original reactor for application of the electric field to a catalyst bed. Schematic images of reactor are portrayed in figure S6. We inserted a thermocouple into the reactor to measure the catalyst bed temperature. In addition, a hand-made Teflon cell was used to conduct DRIFTS measurements during application of the electric field to the catalyst bed. A schematic image of the Teflon cell for *in-situ* DRIFTS measurements is presented in figure S2.

Screening tests and activities for ammonia synthesis over 9.9wt%Cs/5.0wt%Ru/$SrZrO_3$ catalyst in the electric field. We chose 9.9wt%Cs/5.0wt%Ru/$SrZrO_3$ catalyst for detailed investigation because that catalyst showed the highest performance in our screening tests, as presented in table S4. These screening tests were conducted under the same conditions, at 473 K, 0.1 MPa, 200 mg catalyst, 6 mA current, and gas flow (N_2 = 60 SCCM, and H_2 = 180 SCCM). As Figure S13 shows, 9.9wt%Cs/5.0wt%Ru/$SrZrO_3$ catalyst showed a stable activity for 5 h. We conducted screening tests for catalyst support in terms of the ability of proton conduction [48-50]. Also, the property of not only protonic but also mixed ionic and electronic conductor is considered to be significant because electron should run through the catalyst support during the electric field application, proton hopping at the surface of the catalyst. According to the numerous researches, $SrZrO_3$-based oxides show a mixed conductivity with its defect chemistry, even under dry and low temperature conditions [51-53]. Therefore, $SrZrO_3$-based catalyst is considered to show the stable and high activity among these catalysts.

XRD patterns for each support are presented in figure S9. The crystalline structure was almost identical before and after reactions in the electric field. Using Ru catalyst, Cs is known as a promoter, which shows the electron donor effects [5]. Our tests showed that the best amount of Cs is 9.9wt% for 5.0wt%Ru-loaded catalyst. Cs dopant is positively effective to some extent because both the associative reaction and the dissociative one would proceed even when the electric field is applied to catalyst bed at low temperatures. Also, H^+ is partially removed from NH_4^+, which results in producing ammonia thanks to the ability of electron donor for Cs. Furthermore, the activities for ammonia synthesis with or without the electric field at various reaction temperatures, are presented in table S5.

Isotope exchange tests and the calculation for N_2 dissociative rate in the electric field. To avoid the influence of hydrogen poisoning on isotope exchange tests, we conducted isotope exchange tests without the electric field and only supplying N_2 species. Before experiments, hydrogen was

purged at 723 K with Ar = 48 SCCM for 30 min. Then $^{28}N_2$ and $^{30}N_2$ were supplied. These results are presented in figure S1. $^{29}N_2$ was not detected even without hydrogen supply, indicating that the N_2 dissociative rate without the electric field is very low: under the limit of detection.

However, we can calculate the N_2 dissociative rate from the detected outflow rate of $^{29}N_2$. Estimating the reaction mechanism as Langmuir-Hinshelwood mechanism, steady state, and the same zero-point motion energy for ^{14}N and ^{15}N [34, 35], the following equations (1) - (3) are obtained.

Balance equation for N species flow;

$$V_{in} - V_{out} - r_{NH3} / 2 = 0 \quad (1)$$

Equation for N_2 outflow;

$$F_{28} = (F_{28})_0 + V_{out} \cdot (f_{s14} f_{s14}) - V_{in} \cdot (f_{28})_0 \quad (2)$$

$$F_{29} = V_{out} \cdot (2 f_{s14} f_{s15}) = V_{out} \cdot \{2 f_{s14} \cdot (1 - f_{s14})\} \quad (3)$$

In those equations, V stands for the total flow rate, F signifies flow of each species, and f represents the molar fraction of isotopic species. Subscripts $_{28,\ 29,\ 14,\ 15}$ respectively denote $^{28}N_2$, $^{29}N_2$, ^{14}N, and ^{15}N. Also $_s$ signifies the Ru surface. Subscript $_0$ denotes an input value (see also figure S11). From the experiment, we obtained r_{NH3}, F_{28}, $(F_{28})_0$, $(f_{28})_0$, and F_{29}. Then V_{in}, V_{out}, and f_{s14} were calculated. Results of analyses showed that the N_2 dissociative rate, V_{in} or V_{out}, can be calculated as about 36,000 μmol g_{-cat}^{-1} h^{-1} and show f_{s14} as presented in tables S6 and S7.

Influence of metal precursor on ammonia synthesis in the electric field. Our analysis of IR measurements revealed that $NH_4^+Cl^-$ was produced in the electric field. Actually, Cl is regarded as derived from the metal precursor: $RuCl_3$ ($3H_2O$). Therefore, to investigate the influence of Cl on activity, we prepared Cl-free catalyst using metal precursor $Ru(acac)_3$. Table S8 presents activities for both catalysts: Cl precursor and acac precursor. The ammonia synthesis rate, TOF (using CO pulse results, shown in table S3), and the apparent activation energy were nearly equal for the two catalysts. Table S9 and figure S12 present results for isotope exchange tests. The N_2 dissociative rate was calculated using the same procedure for Cl precursor catalyst (table S10). These results demonstrate that both catalysts exhibited almost identical performance, and also that N_2 dissociative rate per unit of electric power was the same value. Therefore, the mechanisms for ammonia synthesis in the electric field with two reactions are regarded as the same. The Cl species is not related to the promotion of ammonia synthesis in the electric field.

Theoretical calculations for ammonia synthesis with/without the electric field on Ru.

From experimental results on the ammonia synthesis on Ru, we considered that two reaction mechanisms are plausible for ammonia synthesis: One is well known as a multistep reaction that is expressed as following elementary steps (10) – (15) in the main text [54], which is named a dissociative mechanism. The other one is designated as an associative mechanism because the association of N_2 and surface H atom takes place before N_2 dissociation, which is expressed as the following elementary steps (16) – (21). Here, the N-N bond dissociation process was assumed to take place from N_2H species**.**

The difference between two mechanisms was investigated using results of theoretical calculations. First, we consider the main reaction sites for ammonia synthesis. Figure 4 and figure S14 show TEM images of Ru particle supported on $SrZrO_3$, and the proposed models of for Ru particle. The figures show that the Ru particle surface consists mainly of Ru(0001), Ru(101) and Ru(100) facets. Among them, Ru(0001), Ru(101) are exposed to the particle surface. Based on this experimentally obtained result, we inferred that the mainly exposed facets of Ru particle are Ru(0001) and Ru(101). The theoretical calculations on these surfaces were considered. The definitions of adsorption sites on Ru(0001) and Ru(101) are presented in figure S10.

Before the investigation on NH_3 synthesis, the effects of electric fields on the catalyst system were examined by making comparison between experimental and theoretical results on *in-situ* IR spectroscopy using CO as probe molecules. The experimental results are presented in figures S3 and S4. As shown in figures S3 and S4, the appearance of the obtained peaks changed into broad ones with the electric field application. If we use each background spectrum (BKG) which was recorded at 523, 573, and 623 K against the obtained spectrum at 473 K in an electric field, then almost the same spectra with the original one (background at 473 K) were obtained, as presented in figure S5. Also as figure S6 shows, the obtained spectra at high temperature around 673 K without an electric field demonstrate that only gas phase of CO peaks were observed due to high temperature. These two results indicate that the change of peak appearance (broad peaks) by the electric field application is not derived from the catalyst bed heating, but from various CO adsorbates. One research group elucidated the polarization effect of catalyst for ammonia synthesis with DC current supply [22]. There might be an analogy, indicating that our Ru catalyst is polarized to some extent during the electric field application, resulting in producing various CO adsorbates (broad peaks). So we estimated the electron state of Ru surface via the peaks of CO adsorbate with/without the electric field. When using Cs-Ru catalyst, the

peak assigned to linear CO around 1986 cm^{-1} [55, 56] shifted to around 2000 cm^{-1}, as shown in figure S3. Moreover, when using Ru catalyst without Cs, the peaks assigned to adsorbed CO on a hollow site around 1750 and 1890 cm^{-1} [57-59] show a clear blue-shift of about 20 – 30 cm^{-1}, as shown in figure S4. This blue-shift with Ru catalyst was more clearly and larger than that with Cs-Ru catalyst. These results implied the effect of Cs addition, which is regarded as an electron donor toward Ru [6-11]. However, the blue-shift of peaks derived from CO vibrational frequency was observed in both cases with/without Cs addition, especially without Cs addition only when the electric field was applied to the catalyst bed. The blue-shift of peaks assigned to adsorbed CO reflected that the electron state of Ru changed by application of the electric field. Previous studies revealed that the peaks assigned to adsorbed CO showed blue shift when Ru was oxidized to some degree [55,56]: this blue-shift was observed when the electron state of Ru became positive.

This blue-shift observed experimentally was confirmed by our theoretical calculations. Table S2 presents the influence of Ru charges on CO adsorbed onto Ru(0001) and Ru(101) by calculations. On Ru(0001), it is widely known that CO preferentially adsorbs on either on-top of hcp three-fold hollow sites [57-59]. Thus, we considered CO adsorptions on these sites. Here, the electric field was expressed by introducing positive and negative charges on the system. Introduction of negative and positive charges on the system induces the elongation and contraction of C-O bond from that of neutral system. For example, the addition of one electrons on the system elongates the C-O bond by 0.008 Å while removing two electron shortens the C-O bond by 0.005 Å. Our calculation have shown that the C-O stretching frequency is also affected by the positive and negative charges. As shown in the Table, the addition and removing of electron in the system induces the decrease and increase of C-O stretching frequency, respectively. For example, ~33 and 35 cm^{-1} decrease and increase of C-O stretch frequency have been observed when adding or removing of one electrons on the system, respectively. Note that these frequency shifts are in accordance with the C-O bond length dependence on positive and negative charges i.e. positive charge induces the contraction on the C-O bond and blue-shift in the spectrum, while negative charge does the CO elongation and red-shift.

Again, the increase in the C-O stretching frequency when removing electrons from Ru-CO system corresponds to the experimentally observed blue shift observed around 1750 - 2000 cm^{-1}. Therefore, we can conclude that the Ru surface under electric field can be expressed by introducing positive charges on or removing electrons from the system.

Next, to elucidate the detailed reaction mechanism of ammonia synthesis under electric field,

we investigated the detailed energetic change of elementary reactions along the reaction path. We consider the ammonia synthesis reaction proceeds via dissociative or associative mechanism. Previous theoretical study by Garden el al. have suggested that the rate-determining step of the dissociative and associative mechanism is the N_2 dissociation and N_2H formation steps, respectively [42].

Based on this model, we carried out geometry optimization of transition state for N_2 dissociation and N_2H formation reactions on Ru. Both (0001) and (101) facets were examined since these facets mainly compose the Ru particle, as observed by the TEM experiment in figure 4. In figure 5, structures of reactant state, transition state, and product state for these reactions were shown. The structures in figure are those of the neutral charge system i.e. without the electric field. We observed no significant change in the structures of reactant state, transition state, and product state between neutral and charged systems. That is, the effect of electric field on the kinetics is mainly via the stability of the surface species and not via the structural factor.

The formation energy (ΔE) and activation barrier (E_a) of these steps are summarized in figures 5 and S7. The effect of electric field was expressed by the positive charge in the system, since our calculation on the CO vibrational frequency have shown that electric field applied to the Ru catalyst bed is well expressed by the positive charge. ΔE in figure 5 indicates that, irrespective of the facets, application of the electric field prevents the N_2 dissociation but promotes the N_2H formation. Without electric field, the N_2H formation was endothermic process. In this case, the reverse reaction i.e. N_2H decomposition becomes faster than the formation. When electric field was applied, on the other hand, N_2H species stably exists on the surface. The E_a plot in figure S7(B) shows similar tendency: the N_2 dissociation becomes slower but the N_2H formation becomes faster under electric field. The amount of the positive charge was changed up to fifteen; this state corresponds to removing ~2 % of valence electron from the whole system since our model system contains ~760 electrons. The decrease of N_2H formation E_a is notable, since it becomes from 1.23 eV to 0.56 eV by the application of electric field.

References for supplementary material

[43] G. Kresse, D. Joubert, *Phys. Rev. B: Condens. Matter.* 1999, **59**(3), 1758.

[44] G. Kresse, J. Furthmuller, *Comput. Mater. Sci.* 1996, **6**(1), 15.

[45] J. P. Perdew *et al. Phys. Rev. Lett.* 1996, **77**(18), 3865.

[46] B. Hammer *et al. Phys. Rev. B: Condens. Matter* 1999, **59**(11), 7413.

[47] J. P. Perdew *et al. Condens. Matter* 1992, **46**(11), 6671.

[48] N. Sata *et al. Solid State Ionics **97***, 437-441 (1997).

[49] S. Shin *et al. Solid State Ionics **40/41***, 910-913 (1990).

[50] T. Hibino *et al. Solid State Ionics **57***, 303-306 (1992).

[51] J. Muller *et al. Solid State Ionics. **97***, 421-427, (1997).

[52] J. A. Labrincha *et al. Solid State Ionics. **61***, 71-75, (1993).

[53] Gemma Heras-Juaristi *et al. J. Power. Source. **331,*** 435-444, (2016).

[54] C. J. Zhang *et al. J. Chem. Phys. **115*** **(2)**, 609-611 (2001).

[55] S. Y. Chin *et al. J. Phys. Chem. B **110***(**2**), 871-882 (2006).

[56] G. H, Yokomizo *et al. J. Catal. **120***, 1-14 (1989).

[57] F. Abild-Pedersen, M. P. Andersson. *Surf. Sci. **601***(**7**), 1747-1753 (2007).

[58] G. Michalk *et al. Surf. Sci. **129***(**1**), 92-106 (1983).

[59] H. Over *et al. Phys. Rev. Lett. **70***(**3**), 315-318 (1993).

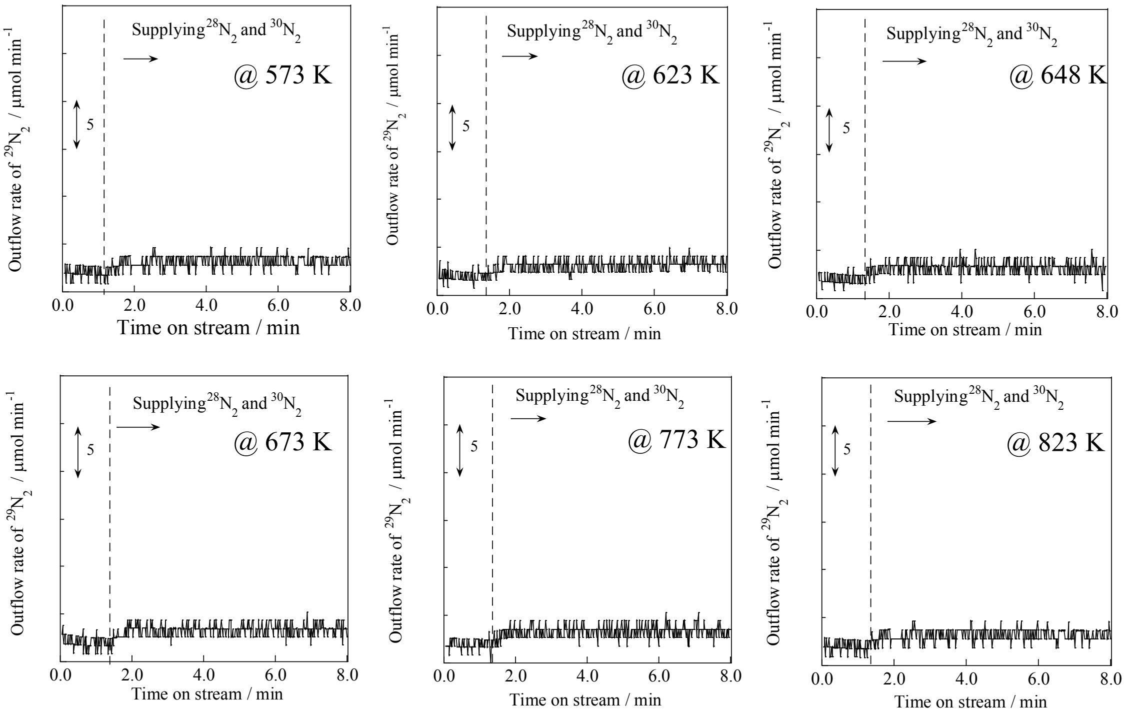

Supplementary Figure S1. Isotope exchange tests using $^{30}N_2$ without an electric field at various furnace temperatures. After pre-reduction at 723 K, hydrogen was purged with Ar for 30 min. Then the temperature was set for each test: catalyst, 9.9wt%Cs/5.0wt%Ru/SrZrO$_3$, 200mg; flow, $^{28}N_2$: $^{30}N_2$: H_2 : Ar = 6 : 6: 0: 48 SCCM.

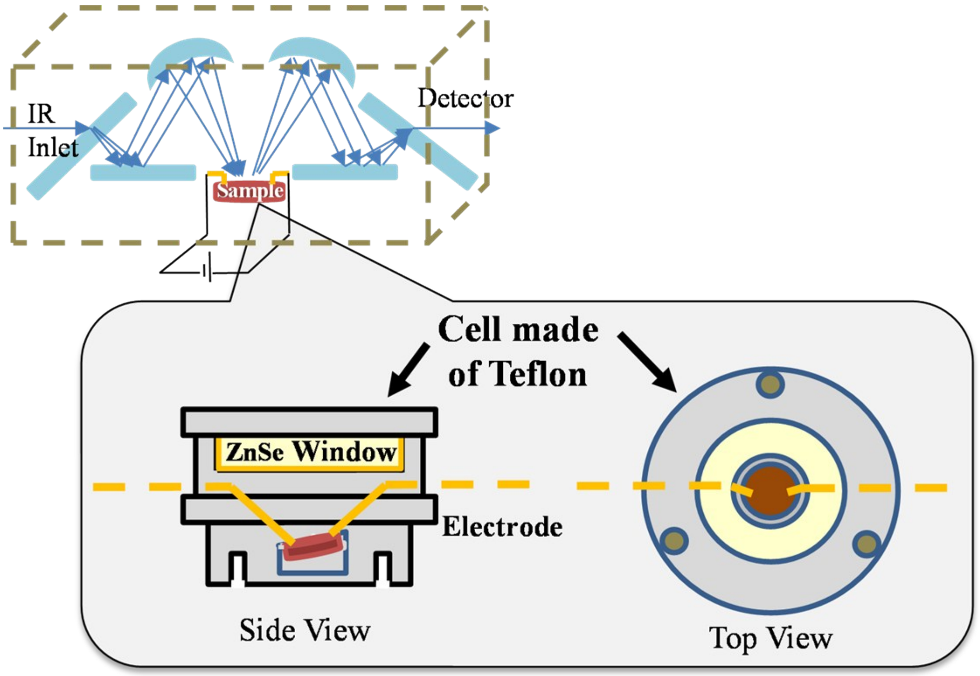

Supplementary Figure S2. Schematic image of Teflon cell for *in-situ* DRIFTS measurements.

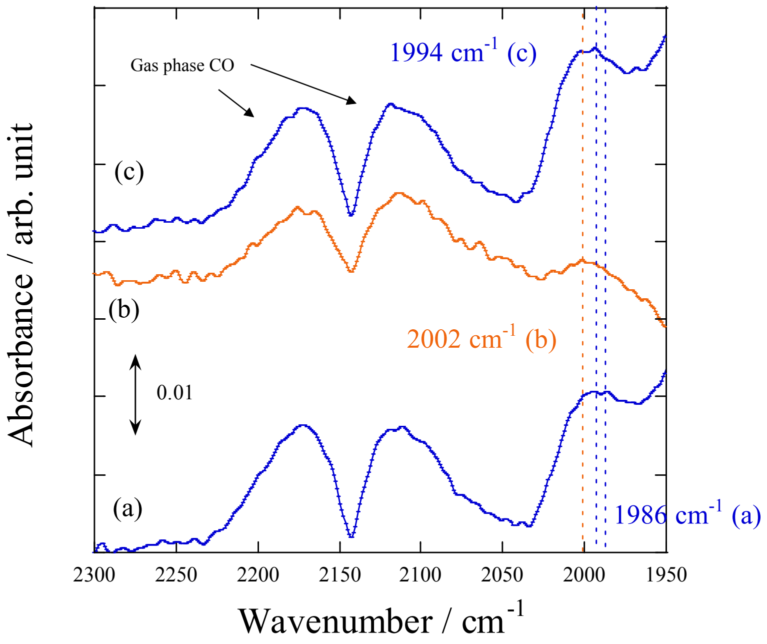

Supplementary Figure S3. *In-situ* IR spectra with CO: (a) before applying EF (b) with EF (6 mA, 0.58 – 0.61 kV): (c) after stopping EF; temperature, 473 K; catalyst, 9.9wt%Cs/5.0wt%Ru/$SrZrO_3$; flow, N_2 : H_2 : CO = 15 : 45 : 1 SCCM; current, 0 or 6 mA.)

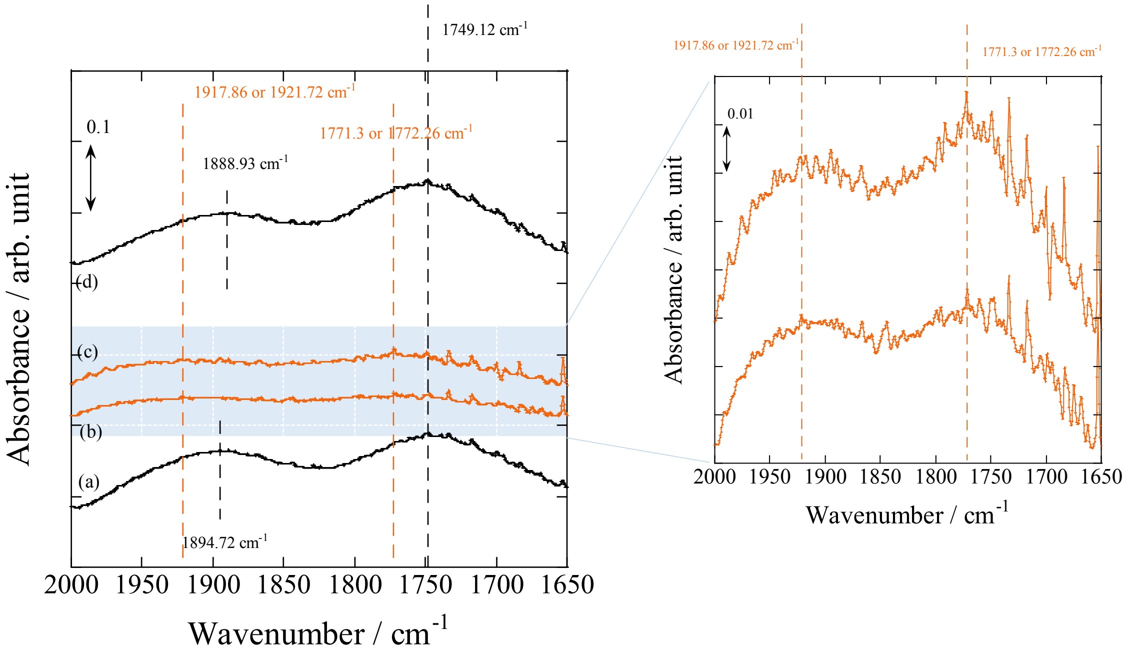

Supplementary Figure S4. *In-situ* IR spectra with CO: (a) before applying EF; (b) with EF 5 min (6 mA, 0.49 – 0.52 kV); (c) with EF 10 min (6 mA, 0.50 – 0.60 kV); (d) after stopping EF; temperature, 473 K; catalyst, 5.0wt%Ru/$SrZrO_3$; flow, N_2 : H_2 : CO = 15 : 45 : 1 SCCM; current, 0 or 6 mA.

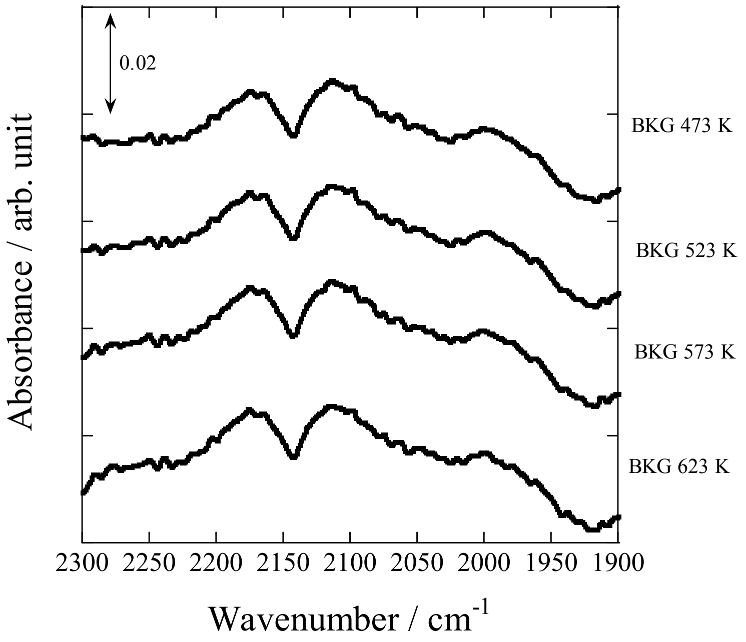

Supplementary Figure S5. *In-situ* IR spectra with various background (BKG) temperature; catalyst, 9.9wt%Cs/5.0wt%Ru/$SrZrO_3$; furnace temperature, 473 K; flow, N_2 : H_2 : CO = 15 : 45 : 1 SCCM; current, 6 mA.

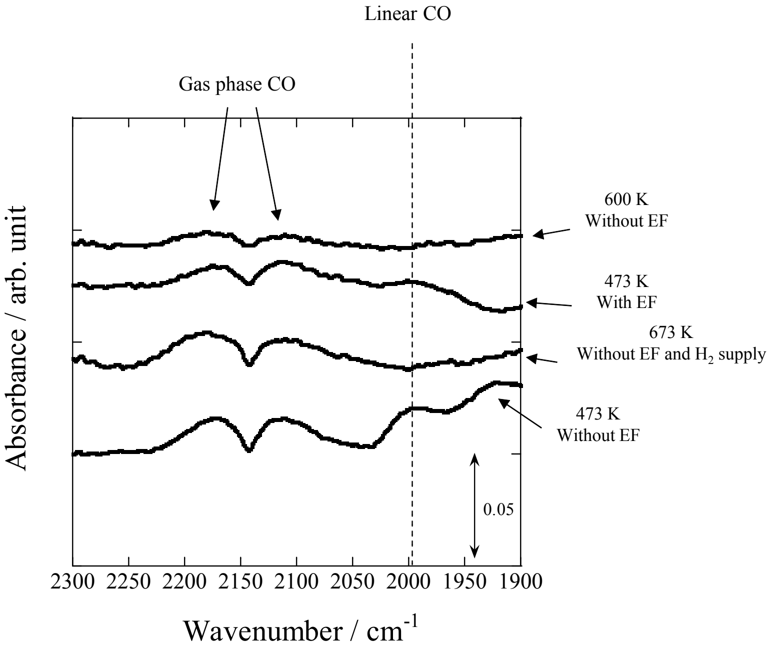

Supplementary Figure S6. *In-situ* IR spectra under various conditions; catalyst, 9.9wt%Cs/5.0wt%Ru/$SrZrO_3$; flow, N_2 : H_2 : CO = 15 : 45 or 0 : 1 SCCM; current, 0 or 6 mA.

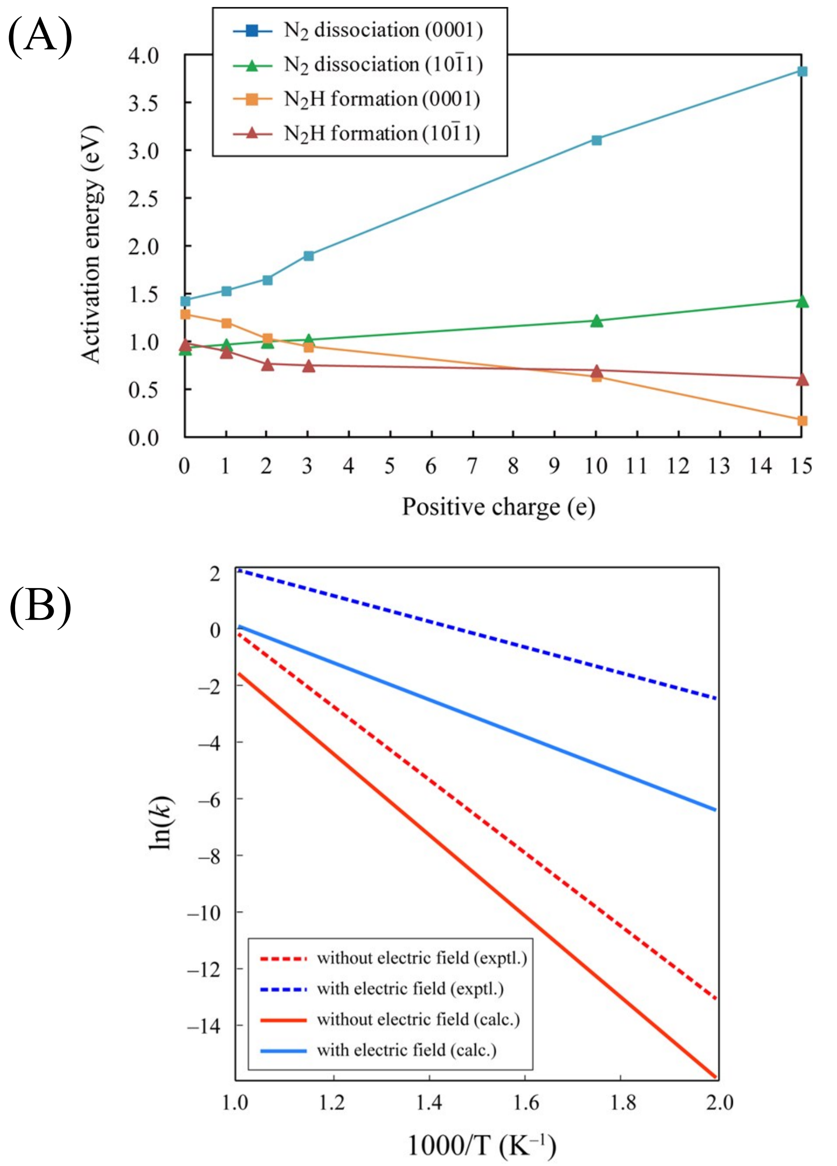

Supplementary Figure S7. Theoretical calculations for ammonia synthesis with/without an electric field. (A) Activation energy of N_2 dissociation and N_2H formation reactions on Ru(0001) and Ru(1011), and its dependence on the electric field expressed by the positive charge. Nudged elastic band method was employed. (B) Arrhenius plot of ammonia synthesis reaction with and without electric field, estimated both experimentally and theoretically. Pre-exponential factor of experimental Arrhenius plot was estimated from Eqs.(19) and (20) for with/without electric field reactions, respectively. Activation energy of fifteen positive charges was used in the computational Arrhenius plot with electric field. Values for Ru(1011) was taken.

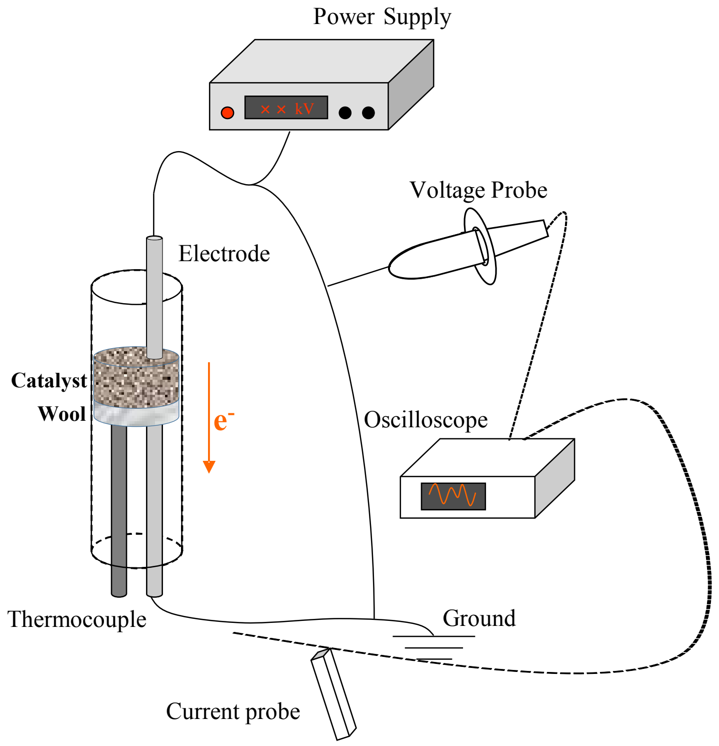

Supplementary Figure S8. Schematic image of the reactor for ammonia synthesis in an electric field.

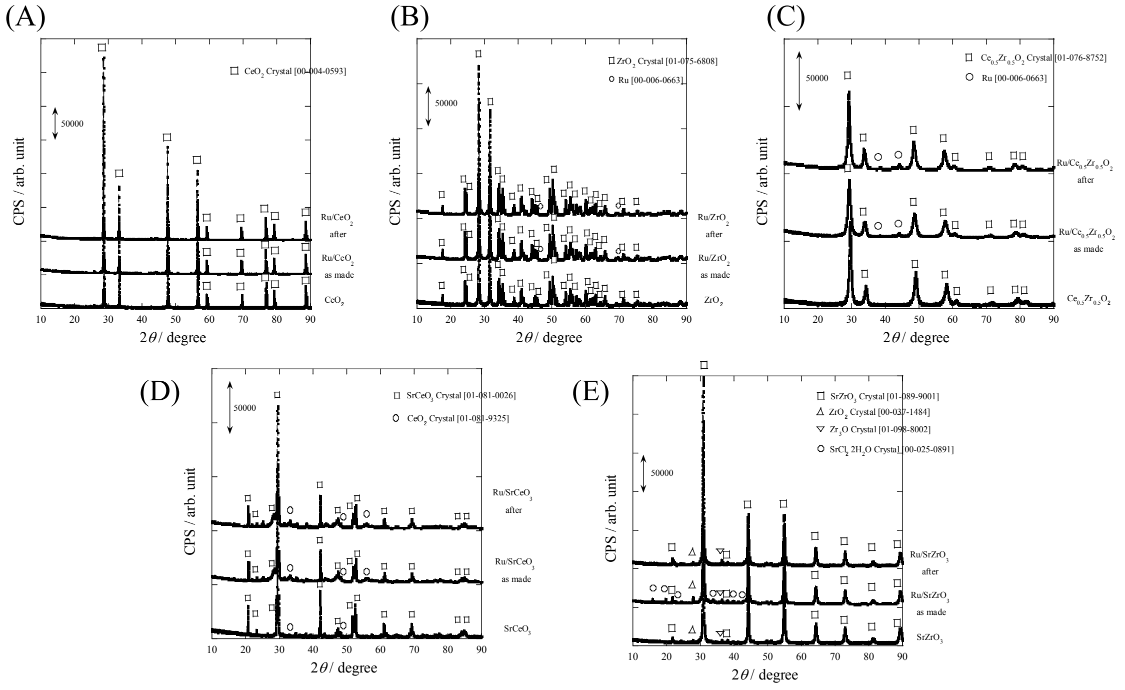

Supplementary Figure S9. XRD patterns for respective catalysts (support only, Ru-load catalyst and after reaction in an electric field): (A) CeO_2 (B) ZrO_2 (C) $Ce_{0.5}Zr_{0.5}O_2$ (D) $SrCeO_3$ (E) $SrZrO_3$

(A)

(B)

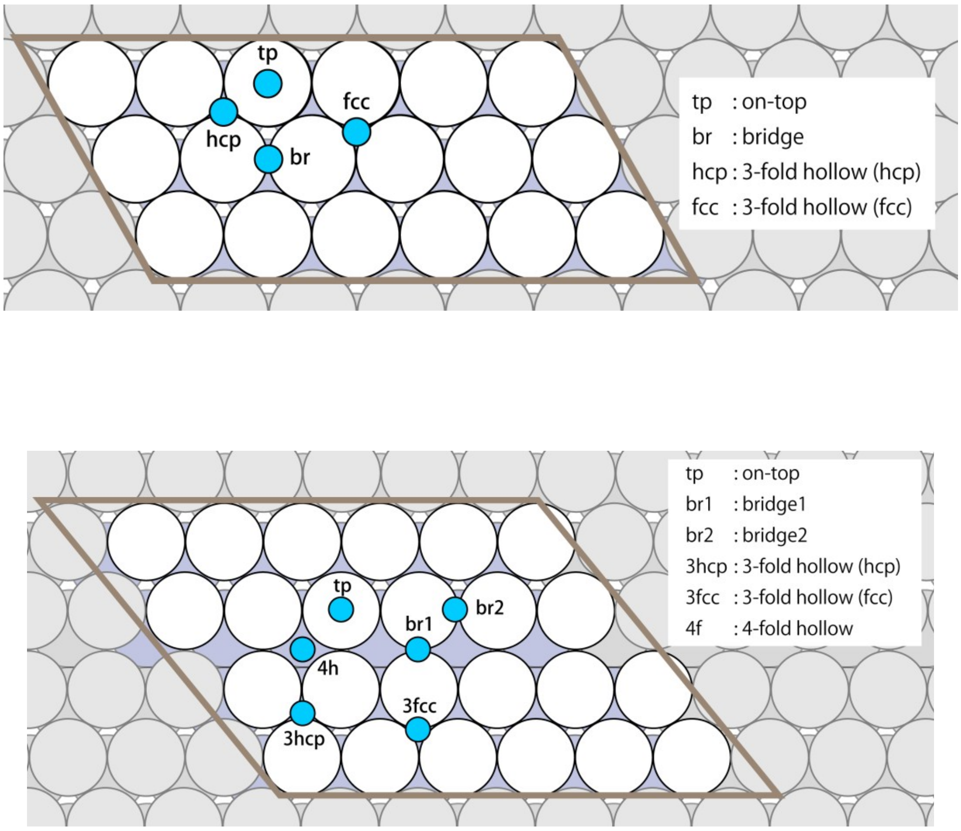

Supplementary Figure S10. Surface model and definition of adsorption sites on Ru: (A) Ru(0001) and (B) Ru(1011).

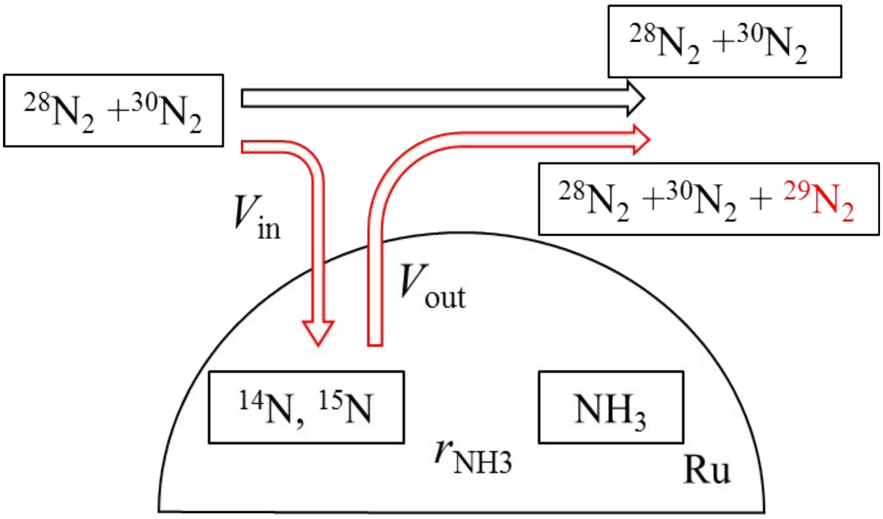

Supplementary Figure S11. Schematic image of the N_2 dissociation process for calculation of the N_2 dissociative rate in an electric field.

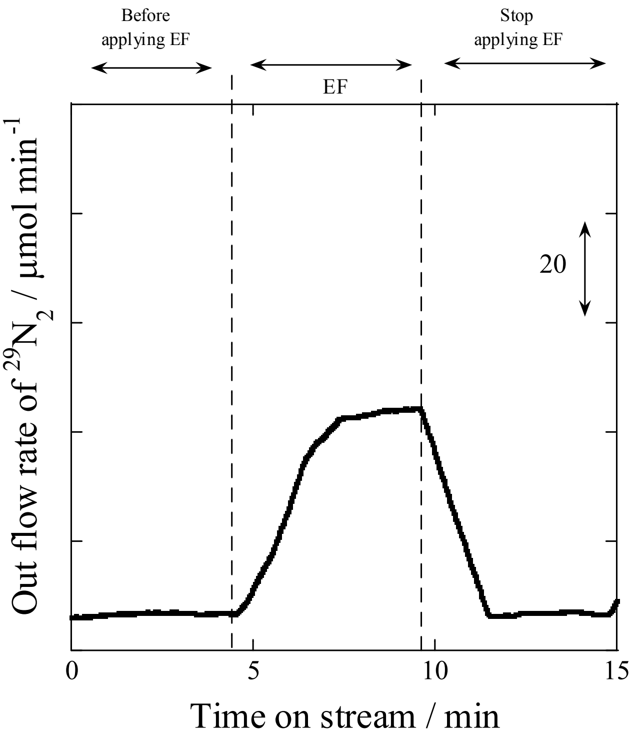

Supplementary Figure S12. Isotope exchange tests using $^{30}N_2$, furnace temperature 473 K, catalyst, 9.9wt%Cs/5.0wt%Ru(acac)/$SrZrO_3$, 200mg; flow, $^{28}N_2$: $^{30}N_2$: H_2 : Ar = 6 : 6: 36: 12 SCCM; current, 0 or 6 mA.

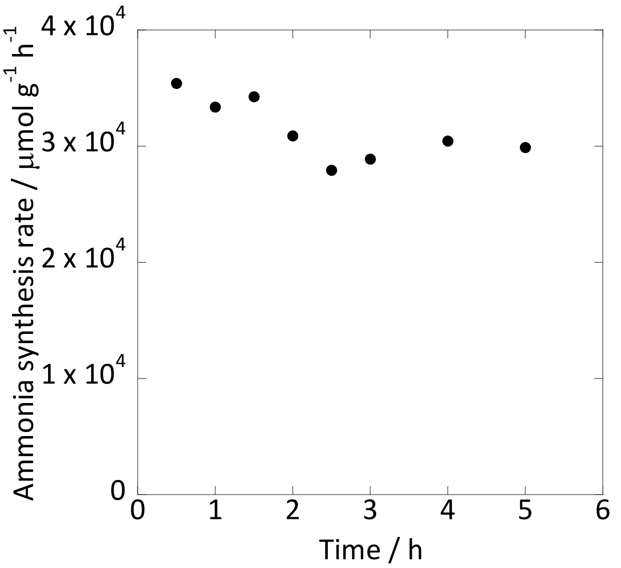

Figure S13. The activity test over 9.9wt%Cs/5.0wt%Ru/$SrZrO_3$ catalyst; catalyst weight, 200 mg; furnace temperature, 473 K; catalyst bed temperature, 624 K; flow, N_2 : H_2 : CO = 60 : 180 SCCM; current, 6 mA.

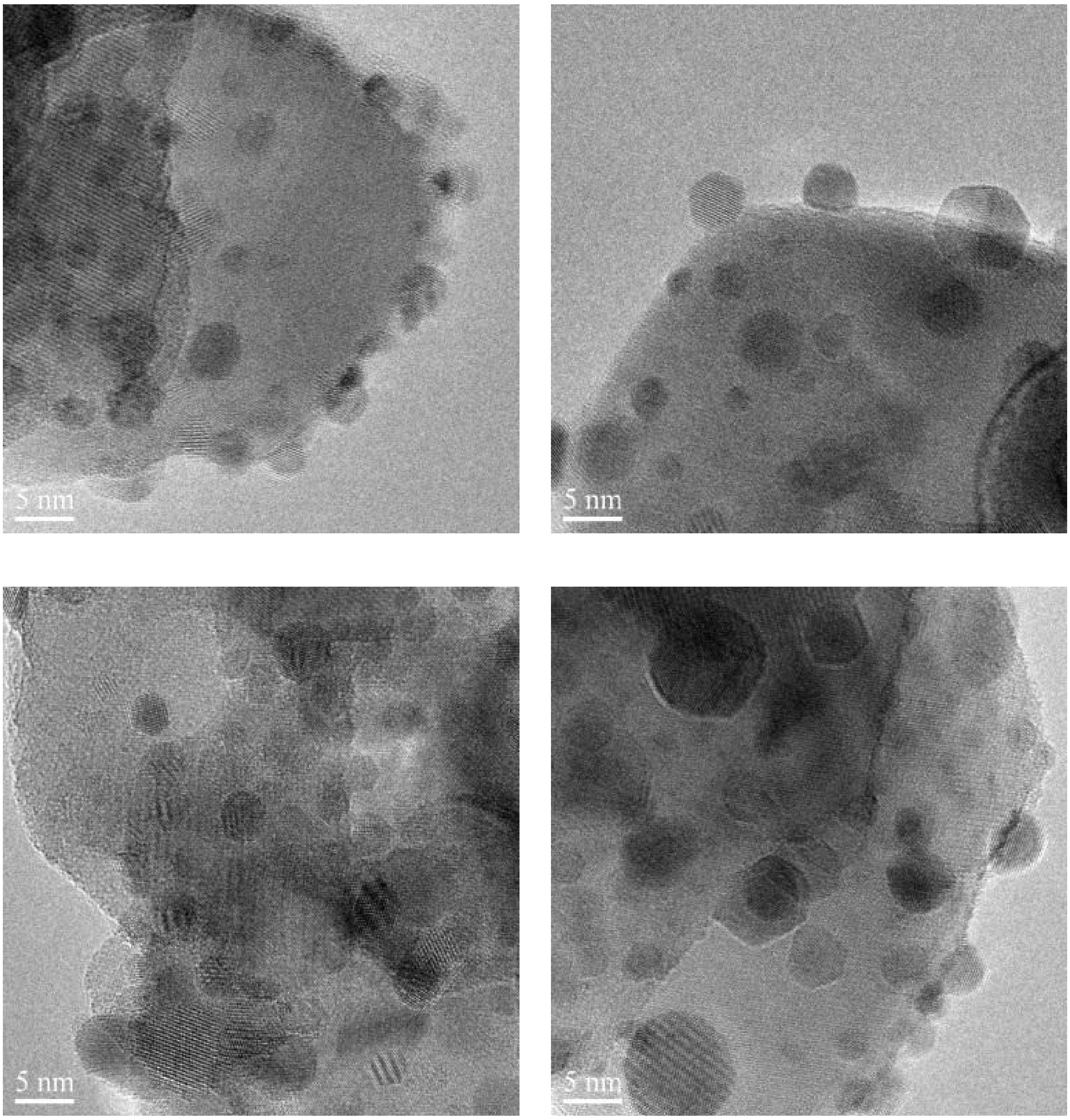

Figure S14. Various TEM images of Ru particle supported on $SrZrO_3$.

Table S1. Kinetic data for analyzing N_2, H_2, and NH_3 pressure dependency of ammonia synthesis rate: catalyst, 9.9wt%Cs/5.0%Ru/$SrZrO_3$, 200 mg; flow, 220 - 280 SCCM, H_2/N_2 = 3 for NH_3 pressure; 240 SCCM with He as a balance gas for N_2 and H_2 pressure; current, 0 or 6 mA.

Condition	$1/q$ h mol^{-1}	P_{N2} or P_{H2} / atm	Furnace Temperature / K	$T_{thermocouple}$ / K	Ammonia synthesis rate / μmol g$_{-cat}^{-1}$ h^{-1}	Voltage / kV	y_0 10^{-4} /-	c 10^{-4} / mol h^{-1}	Orders $r = k P_{N2}^{\alpha} P_{H2}^{\beta} P_{NH3}^{\gamma}$
Without electric field (NH_3)	1.45	*Const.* (P_{N2} = 0.25 atm, P_{H2} = 0.75 atm)	623	634	2163	-	6.29	-	-0.1 (γ)
	1.58			637	2250		7.07		
	1.78			636	2220		7.91		
	1.93			634	2109		8.13		
With electric field (NH_3)	1.44		473	547	575	-0.22	1.67	-	-0.26 (γ)
	1.66			542	567	-0.21	1.89		
	1.81			545	543	-0.22	1.95		
	2.00			545	541	-0.22	2.17		
Without electric field (N_2)	1.74	0.20	623	635	1922	-	6.68	2.04	0.68 (α)
	1.72	0.24		634	2129		7.30	2.28	
	1.72	0.30		633	2484		8.55	2.70	
	1.73	0.35		635	2738		9.47	3.01	
With electric field (N_2)	1.72	0.24	473	532	599	-0.20	2.06	0.167	0.24 (α)
	1.72	0.27		533	601	-0.18	2.07	0.168	
	1.71	0.37		530	647	-0.20	2.21	0.184	
Without electric field (H_2)	1.73	0.58	623	635	2173	-	7.39	2.33	-0.21 (β)
	1.74	0.62		634	2075		7.20	2.29	
	1.74	0.69		635	2131		7.20	2.22	
	1.74	0.77		635	2052		7.14	2.19	
With electric field (H_2)	1.74	0.58	473	544	698	-0.23	2.46	0.204	0 (β)
	1.75	0.63		543	690	-0.23	2.40	0.200	
	1.74	0.70		544	706	-0.23	2.46	0.206	
	1.76	0.79		545	683	-0.23	2.37	0.197	

Table S2. C-O bond length (d_{CO}, in Å), and C-O stretching frequency (v_{CO}, in cm^{-1}) of CO adsorbed on Ru(0001) and Ru(1011).

Surface	Property	Adsorption site	Charge −3	−2	−1	0	1	2	3
Ru(0001)	d_{CO} (Å)	on-top	1.183	1.180	1.173	1.165	1.161	1.156	1.152
		hcp	1.218	1.215	1.208	1.202	1.195	1.189	1.184
		fcc	1.212	1.208	1.200	1.194	1.189	1.182	1.177
	v_{CO} (cm^{-1})	on-top	1855.8	1877.0	1919.5	1953.4	1988.6	2018.1	2048.2
		hcp	1608.3	1630.8	1668.6	1699.9	1734.0	1765.5	1790.3
		fcc	1645.7	1666.4	1711.3	1742.4	1772.9	1812.3	1840.4
Ru(1011)	d_{CO} (Å)	on-top	1.183	1.179	1.174	1.170	1.166	1.162	1.159
		bridge1	1.186	1.182	1.178	1.174	1.170	1.165	1.162
		hcp	1.213	1.208	1.203	1.198	1.194	1.189	1.185
		fcc	1.200	1.194	1.189	1.185	1.180	1.176	1.171
		4-fold	1.232	1.227	1.224	1.217	1.214	1.210	1.206
	v_{CO} (cm^{-1})	on-top	1845.1	1872.5	1905.6	1927.2	1949.5	1967.7	1991.4
		bridge1	1798.7	1812.8	1831.6	1848.1	1869.5	1899.3	1925.5
		hcp	1633.2	1660.3	1688.1	1715.9	1741.3	1766.9	1789.3
		fcc	1626.3	1662.7	1686.5	1710.9	1736.9	1759.4	1785.5
		4-fold	1526.9	1544.5	1566.3	1602.5	1615.7	1634.5	1650.5

Table S3. Dispersion ratio and particle diameter of Ru catalysts analyzed by CO pulse. Loaded Ru, 5.0wt%.

Catalyst	Dispersion ratio / %	Average particle diameter / nm
9.9wt%Cs/Ru (Cl precursor) /$SrZrO_3$	8.13	16.4
9.9wt%Cs/Ru (acac precursor) /$SrZrO_3$	5.56	24.1

Table S4. Activities for ammonia synthesis over various catalysts in an electric field: preset reaction temperature, 473 K; catalyst weight, 200mg; flow, N_2 : H_2 = 60 : 180 SCCM; current, 6 mA; loaded Ru, 5.0wt%.

Catalyst	$T_{thermocouple}$ / K	Voltage / kV	Ammonia synthesis rate per consumption electrical power / μmol g_{-cat}^{-1} J^{-1}
Ru/CeO_2	513	-0.12	0.016
Ru/ZrO_2	613	-0.45	0.032
Ru/$Ce_{0.5}Zr_{0.5}O_2$	594	-0.40	0.062
Ru/$SrCeO_3$	563	-0.29	0.032
Ru/$SrZrO_3$	578	-0.30	0.078
3.3wt%Cs/Ru/$SrZrO_3$	545	-0.24	0.082
6.6wt%Cs/Ru/$SrZrO_3$	549	-0.27	0.074
9.9wt%Cs/Ru/$SrZrO_3$	532	-0.22	0.20
13.2wt%Cs/Ru/$SrZrO_3$	527	-0.22	0.16

Table S5. Activities for ammonia synthesis with or without an electric field: catalyst, 9.9wt%Cs/5.0%Ru/$SrZrO_3$, 200mg; flow, N_2 : H_2 = 60 : 180 SCCM; current, 6 mA.

Pressure / MPa	$T_{thermocouple}$ / K	Voltage / kV	Ammonia synthesis rate / µmol g_{-cat}^{-1} h^{-1}	Apparent activation energy Ea / kJ mol^{-1}
0.1	524	-	38	110
	574		266	
	599		778	
	624		2331	
	633		2737	
0.3	599	-	1247	109
	612		2074	
	624		3211	
	633		4077	
0.5	523	-	38	129
	573		519	
	623		4282	
0.9	573	-	567	121
	598		1232	
	614		3025	
	624		4699	
	634		5537	
0.1	524	-0.44	1415	53
	574	-0.35	4672	
	614	-0.49	8339	
0.5	463	-0.46	2617	32
	527	-0.44	5309	
	574	-0.37	10494	
	627	-0.31	23664	
0.9	474	-0.28	3208	37
	523	-0.35	6453	
	576	-0.43	14614	
	624	-0.47	30099	

Table S6. Isotope exchange tests with or without an electric field.

*The N_2 dissociative rate was calculated from equations (7) - (9).

Condition		Furnace Temperature / K	$T_{thermocouple}$ / K	Voltage / kV	Out flow rate / µmol min^{-1} $^{28}N_2$	$^{30}N_2$	$^{29}N_2$	Ammonia synthesis rate / µmol g_{-cat}^{-1} h^{-1}	N_2 dissociative rate* / µmol g_{-cat}^{-1} h^{-1}
With Electric Field	17 min	473	597	-0.70	251	224	59.2	1742	36000
Without Electric Field	10 min	573	574	-	227	237	Limit of Detection	155	-
	8 min	623	625		217	248		432	
	7 min	648	649		215	248		1003	
	8 min	673	673		214	248		1521	

Table S7. Calculation results of N_2 dissociative rate

V_{in1} / 10^4 µmol g_{-cat}^{-1} h^{-1}	V_{out1} / 10^4 µmol g_{-cat}^{-1} h^{-1}	f_{14} / -
3.65	3.57	0.53

Table S8. Activities for ammonia synthesis with an electric field: catalyst, 9.9wt%Cs/5.0%Ru/$SrZrO_3$, 200mg; flow, $N_2 : H_2$ = 60 : 180 SCCM; current, 6 mA.

Precursor	Furnace Temperature	$T_{thermocouple}$	Voltage	Ammonia synthesis rate	Apparent activation energy Ea	Turn Over Frequency	
	/ K	/ K	/ kV	/ μmol g_{-cat}^{-1} h^{-1}	/ kJ mol^{-1}	10^{-3} / s^{-1}	10^{-3} / J^{-1}
acac	423	505	-0.24	566	31.2	5.7	4.0
	473	542	-0.23	769		7.8	5.6
	523	591	-0.26	1314		13.3	8.5
	573	630	-0.20	1985		20.0	16.7
	598	654	-0.22	3380		34.1	25.9
Cl	373	491	-0.30	306	38.2	2.1	1.2
	473	544	-0.21	537		3.7	3.0
	523	588	-0.22	1001		6.9	5.3
	573	630	-0.21	1865		12.9	10.2
	603	662	-0.21	3621		25.0	20.1

Table S9. Isotope exchange tests with or without an electric field.

*The N_2 dissociative rate was calculated from equations (7) - (9).

Precursor	Time	Furnace Temperature	$T_{thermocouple}$	Voltage	Out flow rate / μmol min^{-1}			Ammonia synthesis rate	N_2 dissociative rate*	N_2 dissociative rate per electric power
	/ min	/ K	/ K	/ kV	$^{28}N_2$	$^{30}N_2$	$^{29}N_2$	/ μmol g_{-cat}^{-1} h^{-1}	/ μmol g_{-cat}^{-1} h^{-1}	/ μmol J^{-1}
Cl	17	473	597	-0.70	251	224	59.2	1742	36000	0.48
acac	9	473	604	-0.56	242	213	43.1	1574	26000	0.43

Table S10. Calculation results of N_2 dissociative rate: for acac precursor

V_{in1}	V_{out1}	f_{14}
/ 10^4 μmol g_{-cat}^{-1} h^{-1}		/ -
2.67	2.59	0.53
